# The paradox-breaking panRAF plus SRC family kinase inhibitor, CCT3833, is effective in mutant *KRAS*-driven cancers

**DOI:** 10.1016/j.annonc.2020.10.483

**Published:** 2021-02

**Authors:** G. Saturno, F. Lopes, I. Niculescu-Duvaz, D. Niculescu-Duvaz, A. Zambon, L. Davies, L. Johnson, N. Preece, R. Lee, A. Viros, D. Holovanchuk, M. Pedersen, R. McLeary, P. Lorigan, N. Dhomen, C. Fisher, U. Banerji, E. Dean, M.G. Krebs, M. Gore, J. Larkin, R. Marais, C. Springer

**Affiliations:** 1 Molecular Oncology Group, Cancer Research UK Manchester Institute, the University of Manchester, Alderley Park, Manchester, UK; 2 Drug Discovery Unit, Cancer Research UK Manchester Institute, the University of Manchester, Alderley Park, Manchester, UK; 3 Gene and Oncogene Targeting Team, CR-UK Cancer Therapeutics Unit, the Institute of Cancer Research, London, UK; 4 Targeted Therapy Team, the Institute of Cancer Research, London, UK; 5 Division of Cancer Sciences, Faculty of Biology, Medicine and Health, the University of Manchester, Manchester, UK; 6 The Christie NHS Foundation Trust, Manchester Academic Health Science Centre, Manchester, UK; 7 The Royal Marsden NHS Foundation Trust, London, UK

**Keywords:** *KRAS*, panRAF/SRC inhibitor, CRC, PDAC, NSCLC

## Abstract

**Background:**

*KRAS* is mutated in ∼90% of pancreatic ductal adenocarcinomas, ∼35% of colorectal cancers and ∼20% of non-small-cell lung cancers. There has been recent progress in targeting ^G12C^KRAS specifically, but therapeutic options for other mutant forms of KRAS are limited, largely because the complexity of downstream signaling and feedback mechanisms mean that targeting individual pathway components is ineffective.

**Design:**

The protein kinases RAF and SRC are validated therapeutic targets in *KRAS*-mutant pancreatic ductal adenocarcinomas, colorectal cancers and non-small-cell lung cancers and we show that both must be inhibited to block growth of these cancers. We describe CCT3833, a new drug that inhibits both RAF and SRC, which may be effective in *KRAS*-mutant cancers.

**Results:**

We show that CCT3833 inhibits RAF and SRC in *KRAS*-mutant tumors *in vitro* and *in vivo*, and that it inhibits tumor growth at well-tolerated doses in mice. CCT3833 has been evaluated in a phase I clinical trial (NCT02437227) and we report here that it significantly prolongs progression-free survival of a patient with a ^G12V^KRAS spindle cell sarcoma who did not respond to a multikinase inhibitor and therefore had limited treatment options.

**Conclusions:**

New drug CCT3833 elicits significant preclinical therapeutic efficacy in *KRAS*-mutant colorectal, lung and pancreatic tumor xenografts, demonstrating a treatment option for several areas of unmet clinical need. Based on these preclinical data and the phase I clinical unconfirmed response in a patient with *KRAS*-mutant spindle cell sarcoma, CCT3833 requires further evaluation in patients with other *KRAS-*mutant cancers.

## Highlights

• We synthesized new drug, CCT3833, that inhibits both RAF and SRC, and so may be effective in *KRAS*-mutant cancers.
• CCT3833 inhibits both signaling pathways *in vitro* and *in vivo*, inhibits tumor growth and leads to regressions in mice.
• CCT3833 significantly prolonged progression-free survival of a patient with a ^G12V^KRAS spindle cell sarcoma.
• CCT3833 is a potential treatment option for several areas of unmet clinical need.

## Introduction

Lung cancer [∼90% of which are non-small-cell lung cancers (NSCLC)] is the most common cancer worldwide and in the UK has a 5-year overall survival (OS) of only 15%.^1^^,^^2^ Colorectal cancer (CRC) is the third most common cancer^1^ and in the UK has a 5-year OS of 60%^2^, and pancreatic ductal adenocarcinoma (PDAC) is the tenth most common cancer in the UK and has the poorest prognosis with 5-year OS of only 5%.^2^ These poor survival rates are partly due to a lack of treatment options. Surgery is the preferable treatment of NSCLC, PDAC and CRC, but most patients present late with inoperable advanced disease and so receive systemic therapy.^3^^,^^4^ Targeted therapies are licensed for NSCLC (*EGFR*, *ALK*, *ROS1* indications) and CRC (*KRAS* wild-type), but *KRAS-*mutated cancer remains an area of unmet clinical need. Critically, *KRAS* is mutated in ∼20% NSCLC, ∼90% PDAC and ∼35% CRC.^5^ These patients receive conventional chemotherapy or immunotherapy, often with limited efficacy and potential toxicity^4^^,^^6^^,^^7^ except for *KRAS-*mutant NSCLC patients who benefit from immune check-point inhibitors compared with *KRAS* wild-type patients.^8^^,^^9^

Thus, although *RAS* (*KRAS*, *NRAS*, *HRAS*) is mutated in ∼25% of all cancers, treatment of these patients is challenging.^10^ Notably, direct inhibitors of KRAS are limited to the p.G12C *KRAS*-mutant,^11^, ^12^, ^13^ so an alternative is to target downstream effectors in the RAF/MEK/ERK pathway, which has led to the development of RAF, MEK and ERK drugs.^14^ However, in *KRAS*-mutant cells, BRAF-selective drugs such as vemurafenib (PLX4032) and dabrafenib^15^ cause paradoxical hyperactivation of the RAF-ERK pathway through formation of BRAF-CRAF homo- and hetero-dimers.^16^ Unfortunately, targeting MEK downstream of RAF with drugs such as trametinib^17^ is ineffective in *KRAS*-mutant cancers because of feedback mechanisms^18^ and adverse side-effects,^19^ and therefore these drugs have been unsuccessful in *KRAS*-mutant PDAC, CRC and NSCLC.^20^^,^^21^

With a pressing need for different approaches for *KRAS*-mutant cancers, here we describe a new drug for this indication. The protein kinase SRC is a master regulator of cancer cell proliferation, metastasis and invasion, and it also potentiates cancer processes such as neo-angiogenesis.^22^ SRC-family kinases (SFKs) are associated with pathogenesis of many cancers, particularly late-stage disease, where its increased activity and expression are associated with disease progression and poorer prognosis. Critically, SRC is a validated target in *KRAS*-mutant CRC,^23^ PDAC^22^^,^^24^ and NSCLC^22^^,^^25^ and it is known that ^G12C^KRAS and SRC inhibitors work synergistically to inhibit ^G12C^KRAS NSCLC cell proliferation.^26^

In this study, we describe CCT3833, a new combined panRAF and SRC inhibitor. We show that CCT3833 does not drive paradoxical activation of the RAF/MEK/ERK pathway in *KRAS*-mutant cells and that its ability to exert dual inhibition of RAF and SRC provides effective therapy in preclinical *KRAS*-mutant PDAC, CRC and NSCLC models. We show that CCT3833 is superior to single-agent panRAF or SRC inhibitors and comparable with combination panRAF + SRC inhibitors in standard two-dimensional tissue culture and more significantly, in three-dimensional spheroids, which are of intermediate complexity between standard monolayer cultures *in vitro* and tumors *in vivo*.

Importantly, CCT3833 has been investigated in a phase I dose-escalation clinical trial (NCT02437227) including 31 patients with solid tumors, of whom at least 10 were known to be *KRAS*-mutant. We report an unconfirmed partial response and prolonged clinical benefit from CCT3833 in one of these patients, diagnosed with a *KRAS*-mutant spindle cell sarcoma. This was the only patient with an unconfirmed partial response on trial. Spindle cell sarcomas are connective tissue tumors characterized by spindle-shaped cells, and are typically treated with anthracyclines, but with limited and variable responses.^27^ Here, we describe a patient with a spindle cell sarcoma presenting a p.G12V KRAS mutation. The patient displayed early disease progression following surgical resection, was not a candidate for doxorubicin chemotherapy and did not respond to the multikinase inhibitor pazopanib. Despite being in the dose escalation phase, CCT3833 achieved a progression-free survival (PFS) of >10 months, and we provide comprehensive analysis of the mechanism of action of CCT3833 in *KRAS*-mutant cancers to reveal how this patient and others could benefit from this agent.

## Materials and methods

### Cell culture

Cell lines were cultured under standard conditions. Human PDAC cell lines (except for MIA-PaCa2) were a gift from Dr Claus Jorgensen, Calu-1 and H460 cells were a gift from Dr Michela Garofalo and H2009 NSCLC cells were a gift from Dr John Brognard. All other human cell lines were from the American Type Culture Collection (ATCC). Short tandem repeat profiles were routinely compared with known ATCC fingerprints and cells were routinely ensured to be mycoplasma free by PCR. Mouse KPC PDAC cells were a gift of Professor Owen Sansom, or were isolated and established in house from transgenic mice as described.^24^ Cells were cultured in Dulbecco's modified Eagle's Medium or RPMI-1640 medium supplemented with 10% fetal bovine serum and 1% penicillin/streptomycin. Short-term growth inhibition assays, long-term cell proliferation assays and tumor spheroid assays were carried out as detailed in Supplementary Methods, available at https://doi.org/10.1016/j.annonc.2020.10.483. A list of the cell lines used and their *KRAS* status is detailed in Supplementary Table S1, available at https://doi.org/10.1016/j.annonc.2020.10.483.

### Mouse allograft/xenograft studies

All animal procedures were carried out in accordance with National Home Office regulations under the Animals (Scientific Procedures) Act 1986 under license PPL-70/7635 and PPL-70/7701 and within guidelines set out by the CRUK Manchester Institute and The Institute of Cancer Research Animal Welfare and Ethical Review Bodies, and described in accordance with Animal Research: Reporting of *In Vivo* Experiments (ARRIVE) guidelines. *In vivo* efficacy and pharmacodynamics studies were carried out as detailed in Supplementary Methods, available at https://doi.org/10.1016/j.annonc.2020.10.483.

### Statistics

For graphs, mean values are shown and error bars represent standard error of the mean unless stated otherwise, (∗) *P* ≤ 0.05 (Student's or Welch's *t*-test as indicated). All *in vitro* experiments were carried out in triplicate unless otherwise stated.

## Results

### CCT3833 is a panRAF plus SFK inhibitor

**Figure 1:**
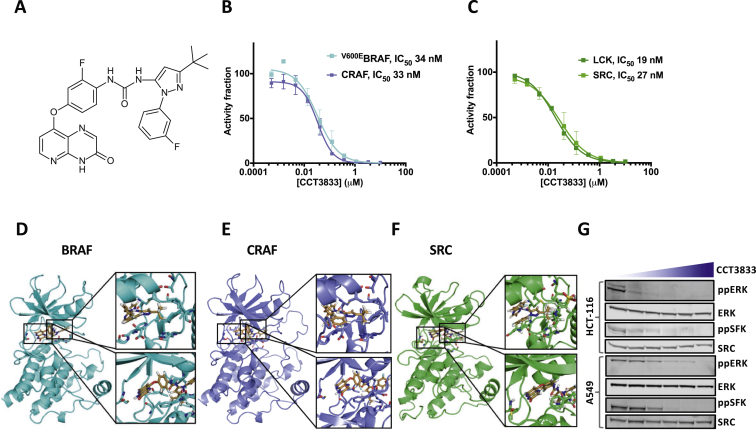
CCT3833 is a panRAF/SRC inhibitor active in *KRAS*-mutant cells. (A) CCT3833 chemical structure. (B) *In vitro* enzyme assay for ^V600E^BRAF or CRAF and (C) SRC or LCK incubated with increasing concentrations of CCT3833. (D) CCT3833 docked into BRAF binding site (pdb4JVG). Inset, detailed CCT3833 interactions with allosteric site (top), ATP binding site (bottom) of BRAF (turquoise). (E) CCT3833 docked into CRAF binding site (homology model derived from pdb4JVG) (orchid). Inset, CCT3833 interactions with allosteric site (top), ATP binding site (bottom). (F) CCT3833 docked into SRC binding site (pdb4AGW) (green). Inset, interactions of CCT3833 with allosteric site (top) and ATP binding site (bottom). (G) Immunoblot for ppERK/ERK and ppSFK/SRC in HCT-116 and A549 cells after 4 h with dimethyl sulfoxide (first lane) or CCT3833 (0.6, 1.2, 2.5, 5, 10 μM respectively). LCK, lymphocyte-specific protein tyrosine kinase; SFK, SRC-family kinase.

CCT3833, (1-[3-tert-butyl-1-[(3-fluoro-phenyl)-1H-pyrazol-5-yl]3-[2-fluoro-4(3-oxo-3,4-dihydropyrido[2,3-b]pyrazin-8-yloxy)phenyl]urea; Figure 1A) is a panRAF inhibitor that inhibits ^V600E^BRAF at 34 nM and CRAF at 33 nM (Figure 1B, Supplementary Table S2, available at https://doi.org/10.1016/j.annonc.2020.10.483). In selectivity screens CCT3833 is mostly inactive against other kinases, with the important exception of SFKs (Supplementary Figure S1A, available at https://doi.org/10.1016/j.annonc.2020.10.483) with SRC inhibition at 27 nM inhibition of the SFK lymphocyte-specific protein tyrosine kinase at 19 nM (Figure 1C, Supplementary Table S2, available at https://doi.org/10.1016/j.annonc.2020.10.483).

Docking studies predict that CCT3833 is a type II inhibitor that binds to the inactive ‘DFG-out’ conformation of BRAF,^28^ and to CRAF and SRC through similar mechanisms (Figure 1D-F). Specifically, the pyridopyrazinone moiety is predicted to interact with the kinase hinge, the central aromatic ring occupies the ATP binding pocket and the pyrazole ring elaborates into an allosteric site created by the DFG moving into the ‘out’ conformation (Figure 1D-F). Moreover, the tert-butyl group is predicted to elaborate into a hydrophobic pocket and the terminal fluoro-substituted phenyl ring points towards the activation loop in all three kinases (Figure 1D-F). These binding similarities indicate how CCT3833 inhibits both RAF and SRC and we confirm that CCT3833 inhibits both ERK (ppERK; downstream of CRAF) and SFK (ppSFK) phosphorylation in a dose-dependent manner in HCT-116 (CRC), A549 (NSCLC) (Figure 1G) and MIA-PaCa2 (PDAC) cells (Supplementary Figure S1B, available at https://doi.org/10.1016/j.annonc.2020.10.483). Our docking studies predict a steric clash would occur between the pyridopyrazinone moiety of CCT3833 and a T338I (the so-called gatekeeper residue) substitution in chicken SRC (Supplementary Figure S2A, available at https://doi.org/10.1016/j.annonc.2020.10.483) and accordingly, we show that CCT3833 inhibits wild-type SRC but not ^T341I^SRC (the human equivalent of T338I) in either HEK-293 or HCT-116 cells (Supplementary Figure S2B-E, available at https://doi.org/10.1016/j.annonc.2020.10.483), supporting our predictions for binding mechanism.

### CCT3833 inhibits KRAS-mutant cancer cell growth

**Figure 2:**
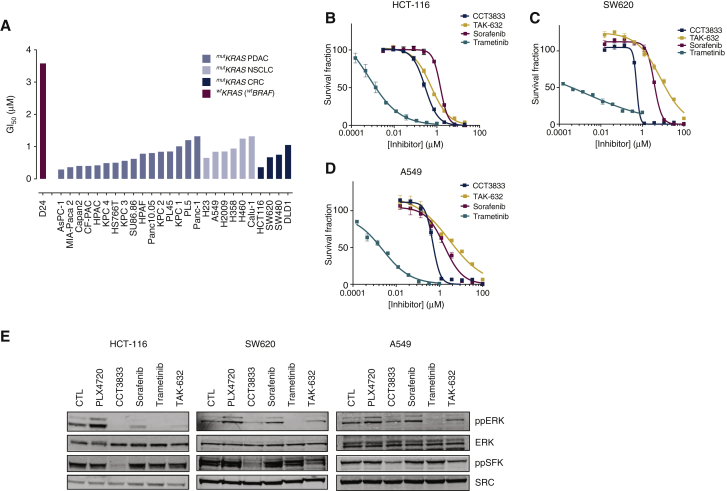
CCT3833 is active in *KRAS*-mutant cells via on target modulation. (A) CCT3833 GI_50_s in *KRAS*-mutant and D24 *WTKRAS* cell lines. (B-D) Proliferation of HCT-116 (B), SW620 (C), A549 (D) cells treated with increasing concentrations of the indicated drugs. (E) Immunoblot for ppERK/ERK and ppSFK/SRC in HCT-116, SW620 and A549 cells after 4 h with dimethyl sulfoxide (DMSO) (CTL), PLX4720, CCT3833, sorafenib, TAK-632 (all at 1 μM), or trametinib (20 nM). SFK, SRC-family kinase.

The data above show that CCT3833 is a panRAF inhibitor that also inhibits SRC. Notably, RAF and SRC are validated targets in *RAS*-mutant cancers, because RAF signals downstream of oncogenic KRAS, and SFKs drive cancer cell proliferation and survival. Accordingly, we show that CCT3833 is active against a panel of *KRAS*-mutant PDAC, CRC and NSCLC cell lines, whereas it is less potent against *KRAS/BRAF* wild-type cells (Figure 2A, Supplementary Table S1, available at https://doi.org/10.1016/j.annonc.2020.10.483). Moreover, compared with other RAF inhibitors, in short-term growth assays CCT3833 inhibits HCT-116 growth more potently than the clinically evaluated panRAF inhibitors TAK-632, ARQ736 and MLN-2480 (Figure 2B, Supplementary Figure S3A, available at https://doi.org/10.1016/j.annonc.2020.10.483). We also show that CCT3833 is more effective than the multikinase inhibitor sorafenib or the BRAF-mutant selective inhibitors PLX4720 and dabrafenib, and that only the MEK inhibitor trametinib is more potent than CCT3833 at inhibiting HCT-116 cells (Figure 2B, Supplementary Figure S3A, available at https://doi.org/10.1016/j.annonc.2020.10.483). We observe similar responses in SW620 (CRC; Figure 2C, Supplementary Figure S3B, available at https://doi.org/10.1016/j.annonc.2020.10.483), A549 (Figure 2D), MIA-PaCa2 (Supplementary Figure S3C and D, available at https://doi.org/10.1016/j.annonc.2020.10.483) and Calu-1 cells (NSCLC, Supplementary Figure S3E, available at https://doi.org/10.1016/j.annonc.2020.10.483), where CCT3833 inhibits growth more effectively than all other RAF inhibitors, with only trametinib being significantly more potent.

Although the panRAF inhibitor TAK-632, and the BRAF inhibitors PLX4720 and dabrafenib inhibit BRAF more potently than CCT3833 in *in vitro* enzyme assays, CCT3833 is more potent at inhibiting *KRAS*-mutant cancer cell growth, so we examine downstream signaling. In HCT-116, SW620, A549, MIA-PaCa2 and Calu-1 cells, PLX4720 induces paradoxical activation of the ERK pathway and although sorafenib and TAK-632 inhibit ppERK, they are less potent than CCT3833 in their ability to do so (Figure 2E, Supplementary Figure S3F-G, available at https://doi.org/10.1016/j.annonc.2020.10.483). Note also that PLX4720, sorafenib and TAK-632 do not inhibit ppSFK in these cells, whereas CCT3833 potently inhibits ppSFK (Figure 2E, Supplementary Figure S3F-G, available at https://doi.org/10.1016/j.annonc.2020.10.483). Finally, although trametinib inhibits ppERK more effectively than CCT3833 in these cells, unlike CCT3833 it fails to inhibit ppSFK (Figure 2E, Supplementary Figure S3F-G, available at https://doi.org/10.1016/j.annonc.2020.10.483).

### RAF and SFK must both be inhibited to block KRAS-mutant cancer growth

**Figure 3:**
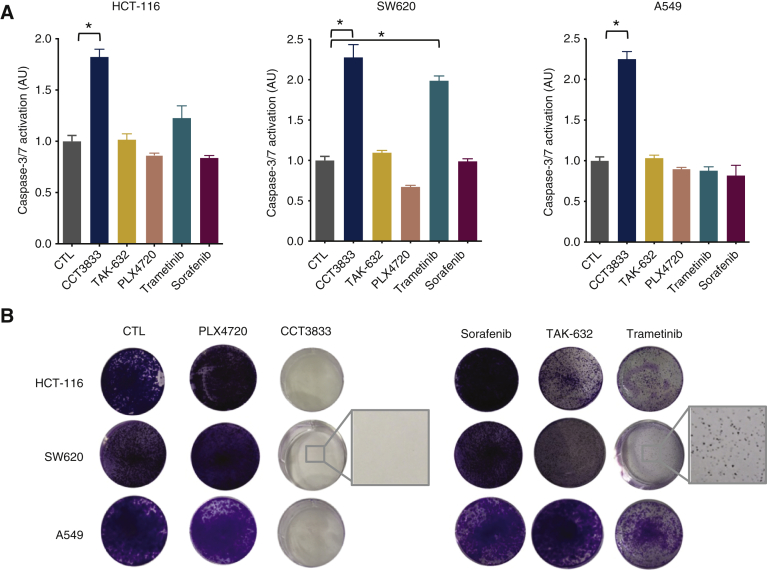
CCT3833 inhibits cell growth and induces apoptosis. (A) Caspase-3/7 activation in HCT-116, SW620 and A549 cells after dimethyl sulfoxide (DMSO) (CTL), CCT3833, TAK-632, PLX4720, sorafenib (all at 1 μM), or trametinib (20 nM), ∗*P* ≤ 0.05 Student's *t*-test. (B) Long-term proliferation assay on HCT-116, SW620 and A549 cells after 9-12 days of treatment with DMSO (CTL), PLX4720, CCT3833, sorafenib, TAK-632 (all at 1 μM), or trametinib (20 nM). Insets are high magnification images of area indicated by squares.

Thus, CCT3833 inhibits both CRAF and SRC in *KRAS*-mutant cancers and so we investigate the contribution of these two activities to the inhibition of long-term cell growth. We show that CCT3833 induces significant caspase-3/7 activation, whereas PLX4720, sorafenib, trametinib and TAK-632 do not activate caspase-3/7 to the same extent (Figure 3A, Supplementary Figure S3H, available at https://doi.org/10.1016/j.annonc.2020.10.483). Accordingly, in long-term clonogenic proliferation assays, only CCT3833 fully inhibits HCT-116, SW620, A549 and MIA-PaCa2 cell growth, whereas colonies are still evident with PLX4720, sorafenib, TAK-632 and also trametinib (Figure 3B, Supplementary Figure S3I and J, available at https://doi.org/10.1016/j.annonc.2020.10.483). These findings are confirmed in two additional human PDAC cell lines (Supplementary Figure S4, available at https://doi.org/10.1016/j.annonc.2020.10.483). Note also that the inhibitors are used at doses to reflect safe plasma exposure achievable *in vivo*. Thus, trametinib is used at 20-30 nM, the maximum tolerated patient plasma concentration,^17^^,^^29^ whereas CCT3833 is used at 1 μM, below the well-tolerated mouse plasma concentration *in vivo* (*vide infra*).

**Figure 4:**
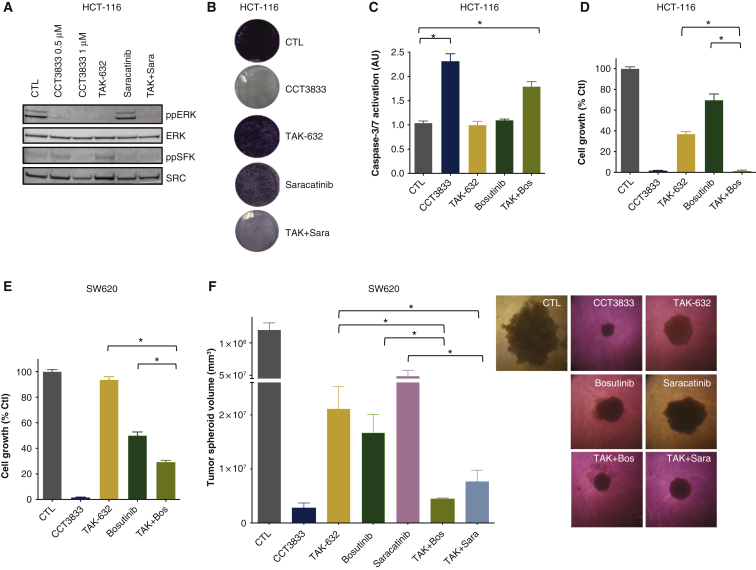
RAF and SRC dual inhibition is required to efficiently inhibit *KRAS*-mutant cell growth. (A) Immunoblot for ppERK/ERK and ppSFK/SRC in HCT-116 cells, 24 h with dimethyl sulfoxide (DMSO) (CTL), CCT3833 (0.5 μM, 1 μM), TAK-632 and saracatinib (both at 1 μM) or TAK-632 plus saracatinib (TAK + Sara; 1 μM each). (B) Long-term proliferation on HCT-116 cells, 10 days, DMSO (CTL), CCT3833, TAK-632, saracatinib (all at 1 μM) or TAK-632 plus saracatinib (TAK + Sara; 1 μM each). (C) Caspase-3/7 activation in HCT-116 cells with DMSO (CTL), CCT3833 (1.2 μM), TAK-632 (1.2 μM), bosutinib (2 μM) or TAK-632 plus bosutinib (TAK + Bos; 1.2 + 2 μM), ∗*P* ≤ 0.05 Student's *t*-test. (D, E) Short-term cell proliferation assays of HCT-116 (D) and SW620 (E) cells treated with CCT3833 (1.2 μM), TAK-632 (1.2 μM), bosutinib (2 μM) or TAK-632 plus bosutinib (TAK + Bos; 1.2 + 2 μM, ∗*P* ≤ 0.05 Student's *t*-test. (F) SW620 spheroids treated with DMSO (CTL), CCT3833, TAK-632 and saracatinib (all 5 μM), bosutinib (1.2 μM) or TAK-632 plus bosutinib (TAK + Box; 5 μM + 1.2 μM) or TAK-632 plus saracatinib (TAK + Sara; 5 μM each) for 5 days. Volume = [(width2 × length)/2], data shown as mean +/− standard deviation, ∗*P* ≤ 0.05 Student's *t*-test. Representative images are shown on the right. SFK, SRC-family kinase.

To assess whether it is necessary to inhibit both ERK and SRC pathways in *KRAS*-mutant cells, we combine known panRAF and SRC inhibitors. As shown earlier, TAK-632 inhibits ppERK but not ppSFK and conversely, we show that the SRC inhibitor saracatinib inhibits ppSFK but not ppERK (Figure 4A). Moreover, together these agents mimic the effects of CCT3833 (Figure 2E) and inhibit both ppERK and ppSFK (Figure 4A). In long-term clonogenic growth assays, neither TAK-632 nor saracatinib alone inhibit colony formation, whereas together they do inhibit colony formation, both in HCT-116 cells and in H23 lung adenocarcinoma cells, again mimicking the effects of CCT3833 alone (Figure 4B, Supplementary Figure S5A, available at https://doi.org/10.1016/j.annonc.2020.10.483). We also assess another SRC inhibitor, bosutinib. Alone, bosutinib does not activate caspase-3/7, but it co-operates with TAK-632 to activate these pro-apoptosis enzymes (Figure 4C) mimicking the effect of CCT3833 alone. Moreover, TAK-632 and bosutinib co-operate to inhibit the short-term growth of HCT-116, SW620 (Figure 4D-E), A549 and MIA-PaCa2 cells (Supplementary Figure S5B, available at https://doi.org/10.1016/j.annonc.2020.10.483), again mimicking the effect of CCT3833 alone. Notably, the combinations of saracatinib plus TAK-632 or bosutinib plus TAK-632 both inhibit the growth of SW620 tumor spheroids similarly to CCT3833 alone, whereas the response to the single agents is significantly less (Figure 4F).

### CCT3833 inhibits KRAS-mutant tumor growth

**Figure 5:**
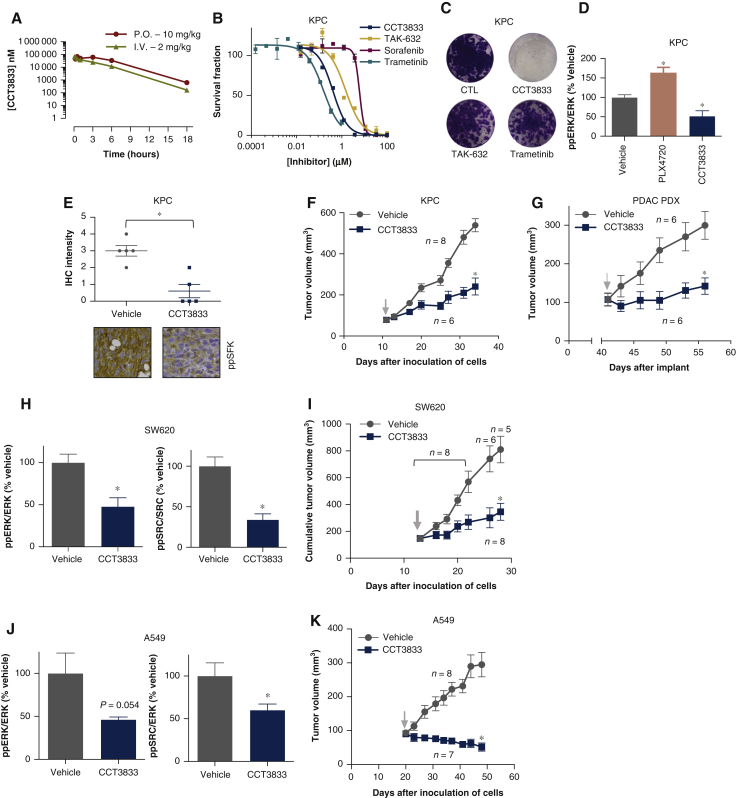
*In vivo* efficacy of CCT3833 in PDAC, CRC and NSCLC. (A) CCT3833 pharmacokinetics studies carried out in BALB/c mice: plasma concentrations at timepoint 18 h show concentration of ∼1 μM when administered by oral gavage. P.O. = oral administration (10 mg/kg); I.V. = intravenous administration (2 mg/kg) in 5% dimethyl sulfoxide (DMSO), 95% water. (B) Proliferation for KPC mouse cells with different drugs. (C) Long-term proliferation of KPC cells, 9 days with DMSO (CTL), CCT3833, TAK-632 (all at 1 μM), trametinib (20 nM). (D) Immunoblot quantification of ppERK/ERK in tumors from the biomarker study in KPC allografts, by oral gavage (p.o.) 4 days with vehicle control (5% DMSO/water), PLX4720 90 mg/kg or CCT3833 (40 mg/kg). See Supplementary Figure S7A, available at https://doi.org/10.1016/j.annonc.2020.10.483, for the blots, ∗*P* ≤ 0.05 Student's *t*-test. (E) Immunohistochemistry and scoring of the ppSFK intensity in tumors from the biomarker study in KPC allografts, ∗*P* ≤ 0.05 Student's *t*-test. Representative images are shown below. (F) Tumor growth curves for KPC allografts, with vehicle control (5% DMSO/water) or CCT3833 (40 mg/kg), p.o., qd, 23 days, ∗*P* ≤ 0.05 Welch's *t*-test on day 23 of treatment. (G) Tumor growth curves for a PDAC PDX, with vehicle control (5% DMSO/water) or CCT3833 (40 mg/kg), p.o., qd, 15 days, ∗*P* ≤ 0.05 Welch's *t*-test on day 15 of treatment. (H) Immunoblot quantification of ppERK/ERK, ppSFK/SRC in SW620 xenografts from biomarker study, p.o., for 4 days with vehicle control (5% DMSO/water) or CCT3833 (40 mg/kg), ∗*P* ≤ 0.05 Student's *t*-test. See Supplementary Figure S7C, available at https://doi.org/10.1016/j.annonc.2020.10.483, for blots. (I) Cumulative tumor growth curves for SW620 xenografts, with vehicle control (5% DMSO/water) or CCT3833 (40 mg/kg), p.o., qd, 15 days, ∗*P* ≤ 0.05 Welch's *t*-test on day 15 of treatment. (J) Immunoblot quantification of ppERK/ERK, ppSFK/ERK in A549 xenografts from biomarker study, p.o., for 4 days, with vehicle control (5% DMSO/water) or CCT3833 (40 mg/kg), ∗*P* ≤ 0.05 Student's *t*-test. See Supplementary Figure S7E, available at https://doi.org/10.1016/j.annonc.2020.10.483, for blots. (K) Tumor growth curves for A549 xenografts, with vehicle control (5% DMSO/water) or CCT3833 (40 mg/kg), p.o., qd, 28 days, ∗*P* ≤ 0.05 Welch's *t*-test on day 28 of treatment. ↓ indicates start of treatment of all tumor growth curves. PDAC, pancreatic ductal adenocarcinoma; PDX, patient-derived xenograft; SFK, SRC-family kinase.

Next, we assess CCT3833 *in vivo*. We show that CCT3833 has good oral bioavailability in mice, excellent pharmacokinetic properties (Figure 5A, Supplementary Table S3, available at https://doi.org/10.1016/j.annonc.2020.10.483), achieves plasma and tumor concentrations well above the GI50 for the target cancer cells and does not accumulate following daily oral doses (Supplementary Table S3, available at https://doi.org/10.1016/j.annonc.2020.10.483, Figure 2A).

We tested CCT3833 in a mouse model of PDAC driven by oncogenic *KRAS* and inactivating mutation of the tumor suppressor TP53 (KPC cells^24^). We confirm, commensurate with our human cell observations, that, CCT3833 is more effective than the other pathway inhibitors apart from trametinib at blocking KPC cell growth in short-term proliferation assays, but only CCT3833 completely abrogates growth of these cells in long-term assays (Figure 5B and C, Supplementary Figure S6A and B, available at https://doi.org/10.1016/j.annonc.2020.10.483). Accordingly, CCT3833 is more potent at inducing caspase-3/7 activation (Supplementary Figure S6C and D, available at https://doi.org/10.1016/j.annonc.2020.10.483). We show CCT3833 blocks ERK and SFK phosphorylation and suppresses tumor growth in KPC cells grown as allografts in mice (Figure 5D-F, Supplementary Figure S7A, available at https://doi.org/10.1016/j.annonc.2020.10.483). Critically, CCT3833 inhibits a human *KRAS*-mutant PDAC patient-derived xenograft (PDX)(Figure 5G, Supplementary Figure S7B, available at https://doi.org/10.1016/j.annonc.2020.10.483).

Importantly, we show that CCT3833 is effective in other human *KRAS*-mutant cells *in vivo*. It inhibits ppERK and ppSFK in SW620 xenografts and suppresses the growth of these CRC tumors in immunocompromised mice (Figure 5H and I, Supplementary Figure S7C and D, available at https://doi.org/10.1016/j.annonc.2020.10.483). Finally, CCT3833 also inhibits ppERK and ppSFK in lung A549 cells and more importantly, at doses that are well tolerated in mice (Supplementary Figure S8, available at https://doi.org/10.1016/j.annonc.2020.10.483), CCT3833 inhibits ERK and SRC, and causes regression of A549 tumors xenografts in mice (Figure 5J and K), and it mediates a significant reduction in the size of foci in the A549 pseudo metastasis tail vein injection model (Supplementary Figure S7E-I, available at https://doi.org/10.1016/j.annonc.2020.10.483).

### CCT3833 improves progression-free survival in a patient with ^G12V^KRAS spindle cell sarcoma

**Figure 6:**
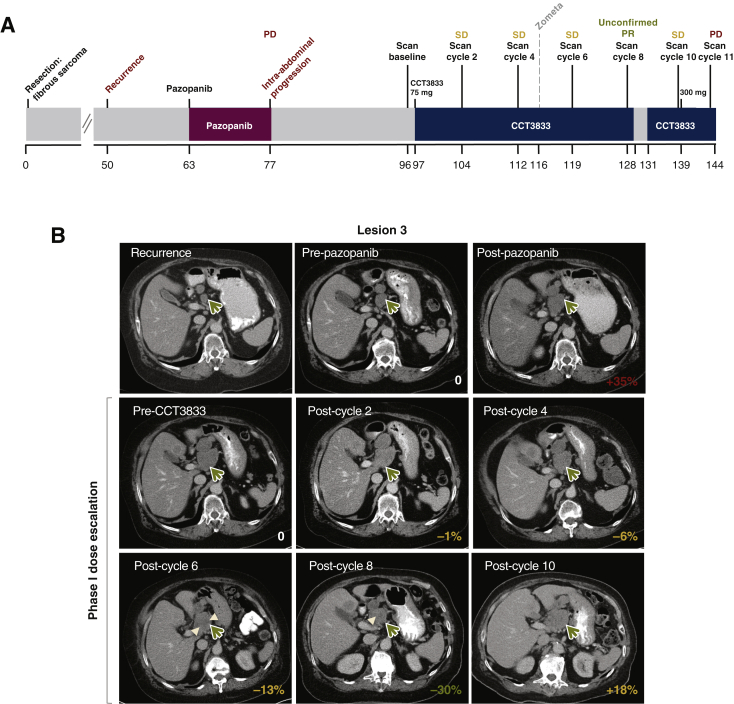
Clinical response in a patient with *KRAS*-mutant spindle cell sarcoma NOS. (A) Patient clinical history. (B) Computed tomogrpahy scans of lesion 3 (mass superior to pancreatic head, green arrow, see also Supplementary Table S4, available at https://doi.org/10.1016/j.annonc.2020.10.483, for blots) of the patient at recurrence, before and after pazopanib treatment; before and during CCT3833 treatment. 0 = baseline (pretreatment) before each treatment; percentage of sum of all marker lesions is reported on the bottom right of each scan as per RECIST 1.1 criteria (Table 1). Hypodensity within the lesion seen on scan at cycle 6 and 8 suggests necrosis due to treatment response (white arrowhead). NOS, not otherwise specified; PD, progressive disease; unconfirmed PR, unconfirmed partial response; SD, stable disease.

A previously fit patient aged in their 70s presented with a 1 year history of non-specific symptoms and was diagnosed with a large intra-abdominal mass associated with the pancreas and invading into the liver parenchyma (Figure 6A). This was resected and histopathological assessment revealed a lobulated tumor composed of ill-defined fascicles of spindle cells, with oval to elongated moderately pleomorphic nuclei, pale eosinophilic cytoplasm and up to 9 mitoses per 10 high powered fields, later classified as a spindle cell sarcoma NOS (not otherwise specified). Immunohistochemistry was diffusely positive for CD34, but negative for other markers including S-100, SOX10, DOG1, CD117, SMA, desmin, AE1-3, EMA, CD21 and CD23. Moreover, the tumor was negative for the NAB2-STAT6 fusion transcripts.

One year after tumor resection, the patient presented with a multifocal intra-abdominal recurrence, which was inoperable. Although it was later diagnosed as spindle cell sarcoma, due to some histopathological features, it was treated as a malignant solitary fibrous tumor. The patient commenced on the multikinase inhibitor pazopanib, but had extensive disease progression at first radiological assessment after only 12 weeks of treatment (Figure 6A and B, Table 1, Supplementary Table S4, available at https://doi.org/10.1016/j.annonc.2020.10.483). The patient was not a candidate for doxorubicin chemotherapy and molecular testing was carried out using a 19-gene MassArray assay (Sequenom Oncocarta v1.0, San Diego, CA), which revealed a *KRAS* C35G>Tp.Gly12Val mutation, so the patient was enrolled on to the phase I trial of CCT3833 (NCT02437227) and was allocated to the lead-in dose CCT3833 (75 mg once a day, continuous dosing). Scans were taken at baseline and every 8 weeks during CCT3833 treatment (Figure 6B). In stark contrast to the progression seen with pazopanib, each of the scans after commencing CCT3833 treatment show stable disease with achievement of an unconfirmed partial response after eight cycles as defined by RECIST 1.1 (Figure 6B, Table 1, Supplementary Table S4, available at https://doi.org/10.1016/j.annonc.2020.10.483). As a non-RECIST progression was seen on imaging after cycle 10, the patient underwent intrapatient dose escalation to 300 mg once daily, but a scan on cycle 11 (Table 1) confirmed RECIST disease progression and the patient discontinued treatment on day 17 of cycle 12.

**Table 1:** Patient scan marker lesion measurements (RECIST version 1.1) at baseline and on treatment.

Weeks from diagnosis	Drug	Cycle	Lesion1	Lesion2	Lesion3	Lesion4	Lesion5	Total (cm)	% Change
62.7	–	0	1.5	2.2	3.0	1.0	0.5	8.2^a^	0
75.7	Pazopanib	3	2.2	2.5	4.0	1.5	0.9	11.1	+35
95.9	–	0	3.3	3.1	4.7	2.1	1.0	14.2^a^	0
104.3	CCT3833	2	2.9	3.7	4.5	2.0	0.9	14.0	−1
112.3	CCT3833	4	2.9	3.4	4.2	2.3	0.5	13.3	−6
118.6	CCT3833	6	2.6	2.7	4.1	2.4	0.5	12.3	−13
128.3	CCT3833	8	1.7	2.2	3.6	2.0	0.5	10.0^b^	−30
138.6	CCT3833	10	1.7	2.7	4.5	2.9	0.0	11.8	+18
143.3	CCT3833	11	1.8	3.0	4.8	3.2	0.0	12.8	+28

a Baseline measurements.

b Nadir.

## Discussion

Herein, we report that the new inhibitor, CCT3833, mediated an unconfirmed partial response in a patient with aggressive *KRAS*-mutant spindle cell sarcoma who was not eligible for surgery or chemotherapy, and who did not respond to the multikinase inhibitor pazopanib. Despite being in the dose-escalation phase of the clinical trial (NCT02437227), at 75 mg p.o. qd continuous dosing of CCT3833, the patient achieved progression-free survival for 8 months, and did not progress until the 12th cycle of treatment. Together with our preclinical data, this indicates that CCT3833 has potential for the treatment of *KRAS*-mutant tumors. Specifically, our preclinical data demonstrate that CCT3833 is effective in *KRAS*-mutant CRC, NSCLC and PDAC due to its dual anti-panRAF plus anti-SRC activity. RAF is a validated target directly downstream of oncogenic RAS, and SRC is also a validated therapeutic target in CRC, NSCLC and PDAC, where it is hyperactivated and drives cell proliferation and metastasis.^22^, ^23^, ^24^, ^25^ Accordingly, SRC inhibitors cooperate with drugs that target the EGFR/RAS pathway.^26^^,^^30^^,^^31^

Our findings that CCT3833 inhibits the growth of *KRAS*-driven murine PDAC *in vitro*, and *in vivo*, are aligned to the literature, validating SRC as a therapeutic target in PDAC, where its overexpression or hyperactivation are markers of poor clinical outcome.^24^ Moreover, SRC inhibitors are active in preclinical PDAC models and achieve minor clinical responses in PDAC patients.^22^ SRC is similarly overexpressed or hyperactivated in CRC^23^ and moreover, *BRAF*-mutant CRC cells can switch between RAF/MEK/ERK and receptor tyrosine kinase signaling,^32^ so cell growth is only prevented when both pathways are inhibited. This plasticity may explain the shorter duration of response to BRAF and MEK inhibitors in CRC^21^ and may also underpin why mutant *KRAS* opposes the antitumor effects of EGFR inhibitors in CRC.^33^ Notably, MEK and EGFR inhibitors cooperate to block EGFR inhibitor-resistant CRC tumor growth,^33^ and we propose therefore that CCT3833 is effective in CRC because it targets the two key pathways downstream from mutant RAS and the hyperactivated receptor tyrosine kinases such as EGFR. Similarly, synergistic efficacy has been shown *in vivo* by inhibiting the MAPK pathway plus SRC in KRAS/PIK3CA double-mutant CRC cells.^34^

Finally, SRC and ERK signaling are both critical for the growth of *KRAS*-mutant NSCLC.^20^^,^^25^ Clinical trials with trametinib in *KRAS*-mutant NSCLC patients, alone or in combination with chemotherapy, show that single agent MEK inhibitors achieve no improvement compared with chemotherapy and that toxicity limits their clinical use in combination.^19^^,^^20^ Again, we posit that CCT3833 is effective in NSCLC because of its ability to simultaneously inhibit SRC and ERK signaling. Critically, CCT3833 mediates tumor regression in ^G12S^KRAS-mutant NSCLC xenografts, so it could be considered for treatment of *KRAS*-mutant NSCLC patients who fail chemotherapy and/or immunotherapy.

In summary, we describe the discovery of CCT3833, a new panRAF/SRC inhibitor, and show that it is effective in *KRAS*-mutant cancer models, because RAF and SRC are central nodes in *KRAS-*mutant cancers. CCT3833 differs from the RAF dimer inhibitor LY3009120^35^ because it also inhibits SRC and is effective in PDAC. We posit that CCT3833 inhibits tumor growth in *RAS*-mutant models through on-target inhibition of BRAF and CRAF, and additional on-target inhibition of SRC. Critically, CCT3833 induces tumor cell death and elicits therapeutic efficacy at well-tolerated doses in mice, and it is evaluated in patients in a phase I clinical trial, achieving a proof-of-concept unconfirmed clinical response in a patient with aggressive *KRAS*-mutant spindle cell sarcoma who was not eligible for other treatments. Taken together, our data support the further clinical evaluation of CCT3833 in patients with *KRAS*-mutant cancers.
