# Supplementary material for: The paradox-breaking panRAF plus SRC family kinase inhibitor, CCT3833, is effective in mutant KRAS-driven cancers
Source: Ann Oncol. 2021 Feb;32(2):269–78. doi: 10.1016/j.annonc.2020.10.483 (PMC7839839; doi:10.1016/j.annonc.2020.10.483)

Saturno et al

Supplementary Figure S1

A

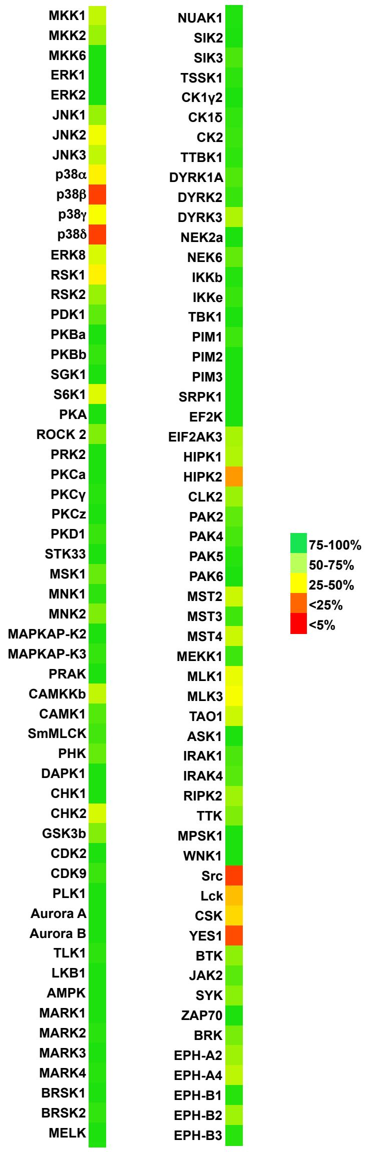

B

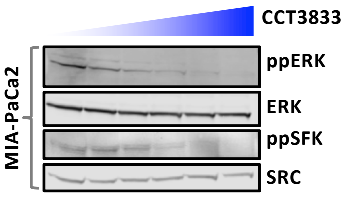

Saturno et al

Supplementary Figure S2

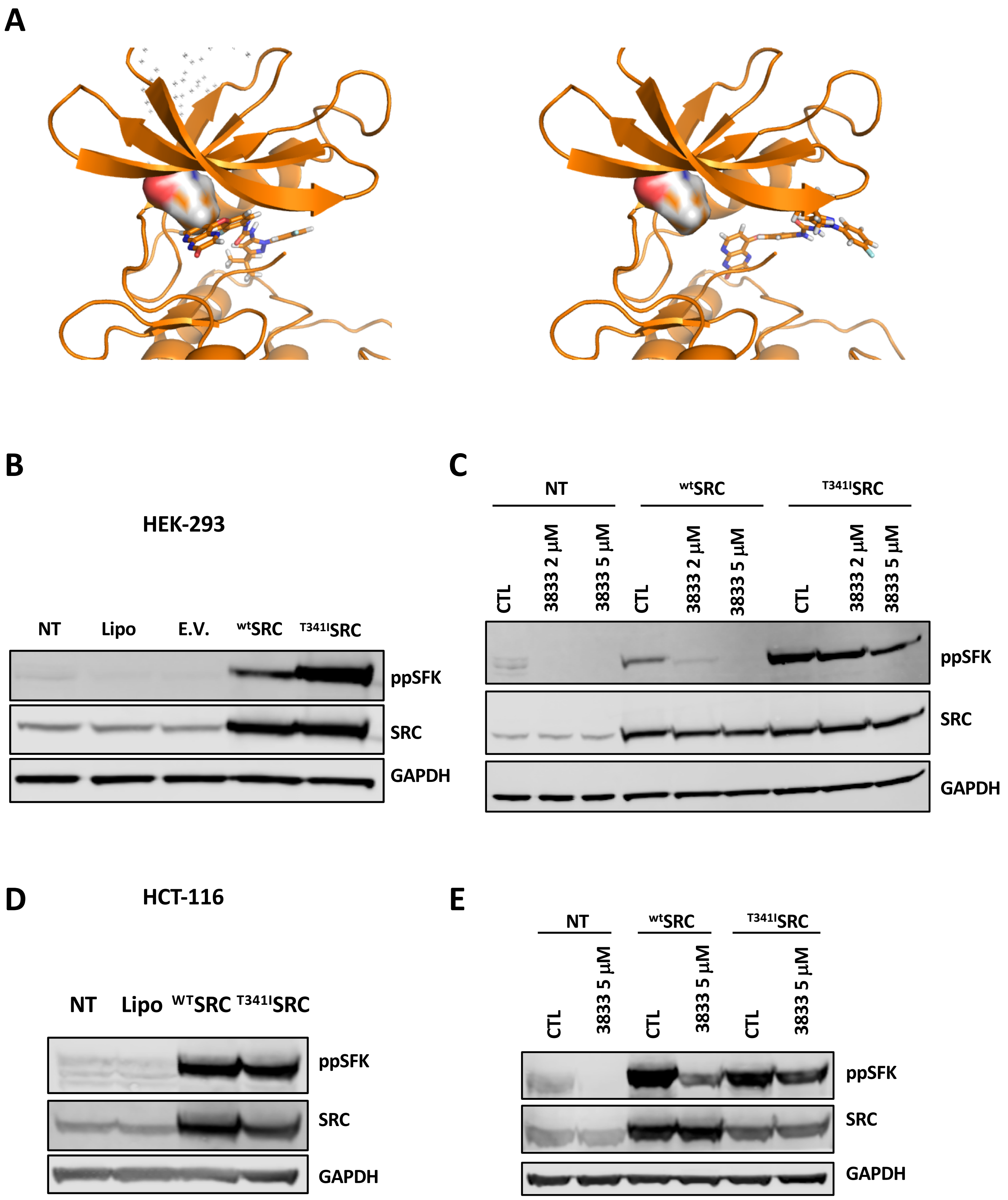

Saturno et al

Supplementary Figure S3

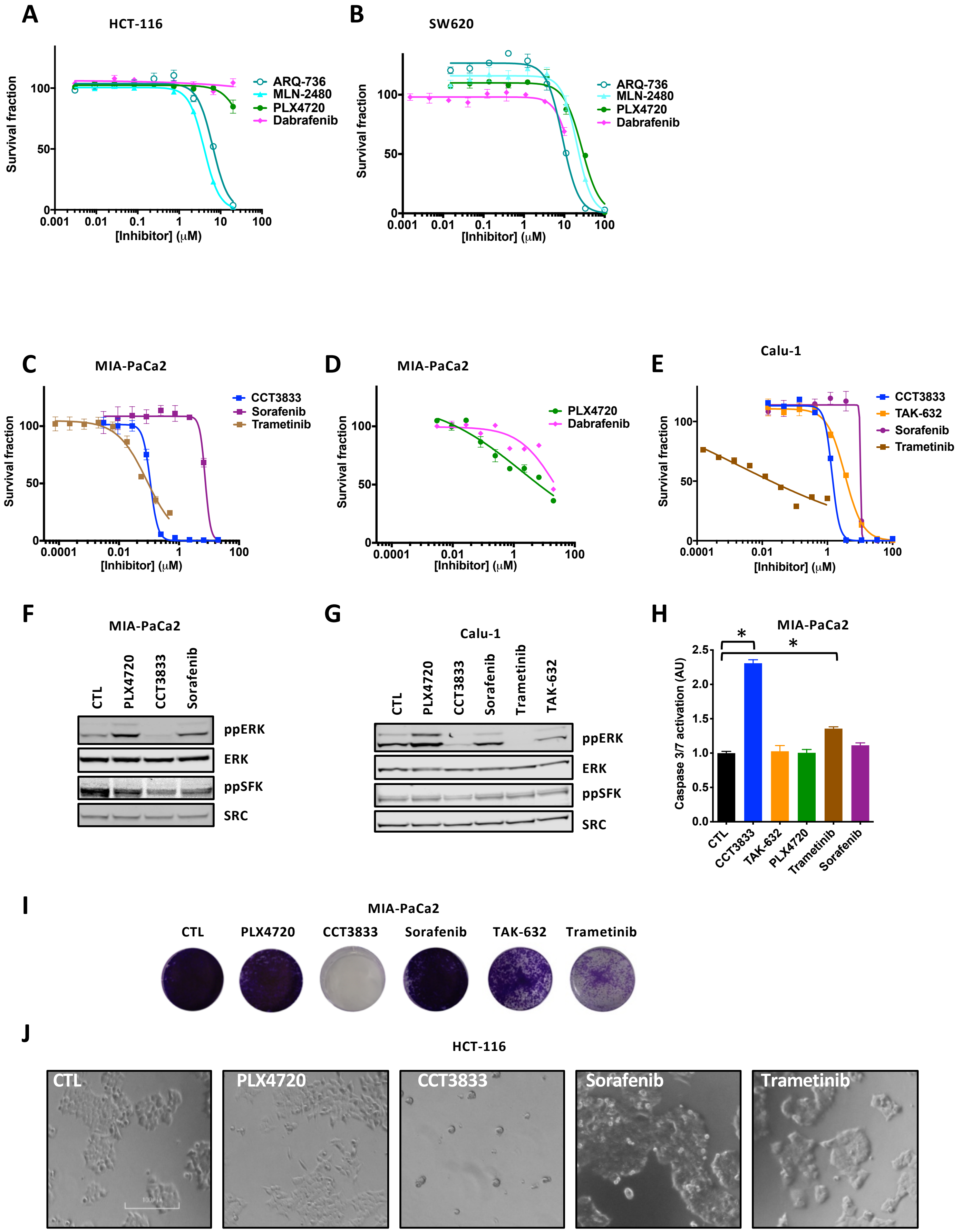

Saturno et al

Supplementary Figure S4

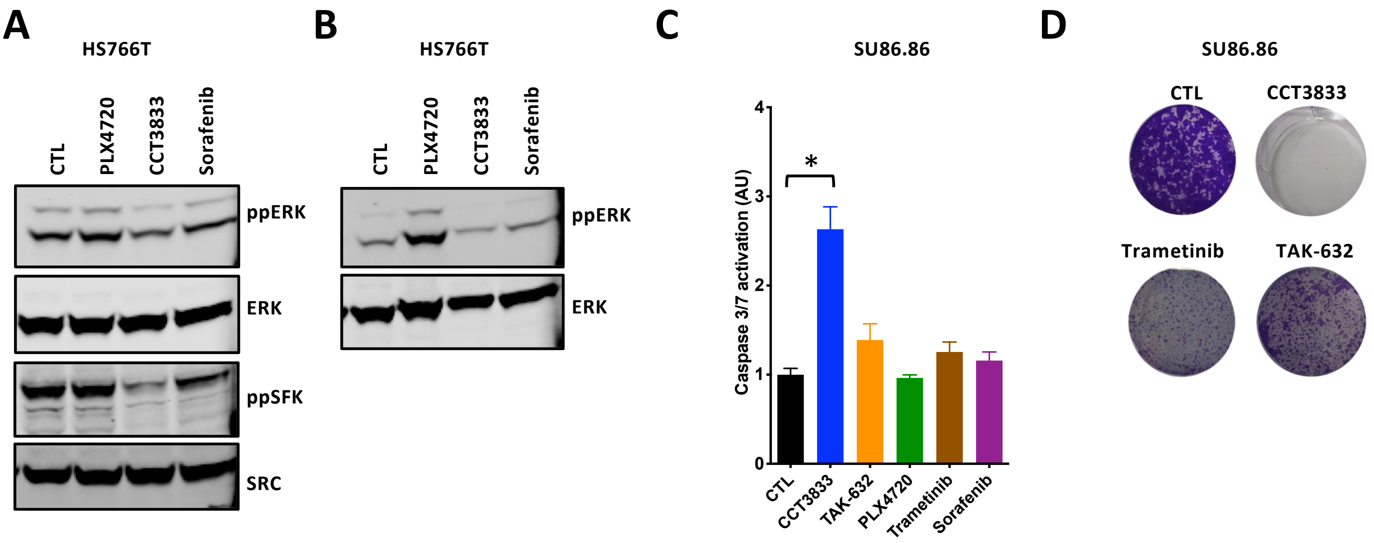

Saturno et al

Supplementary Figure S5

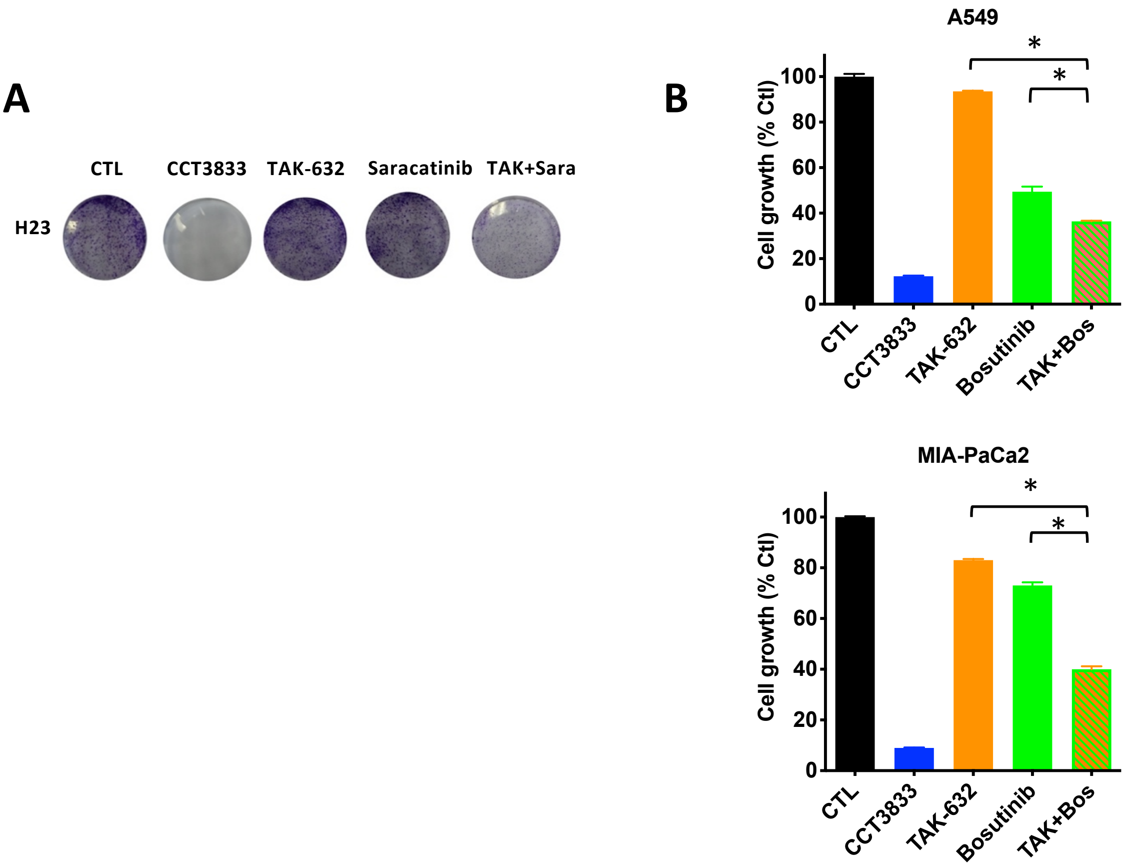

Saturno et al

Supplementary Figure S6

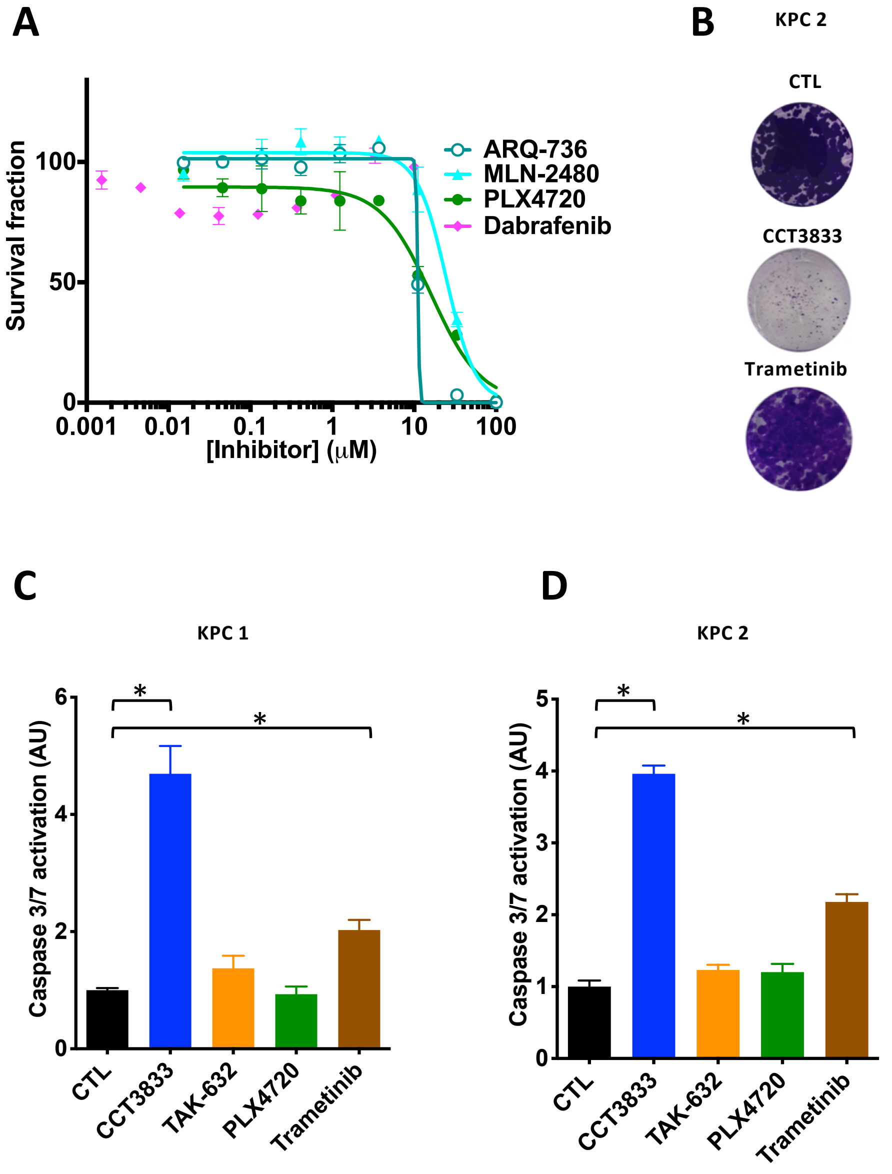

Saturno et al

Supplementary Figure S7

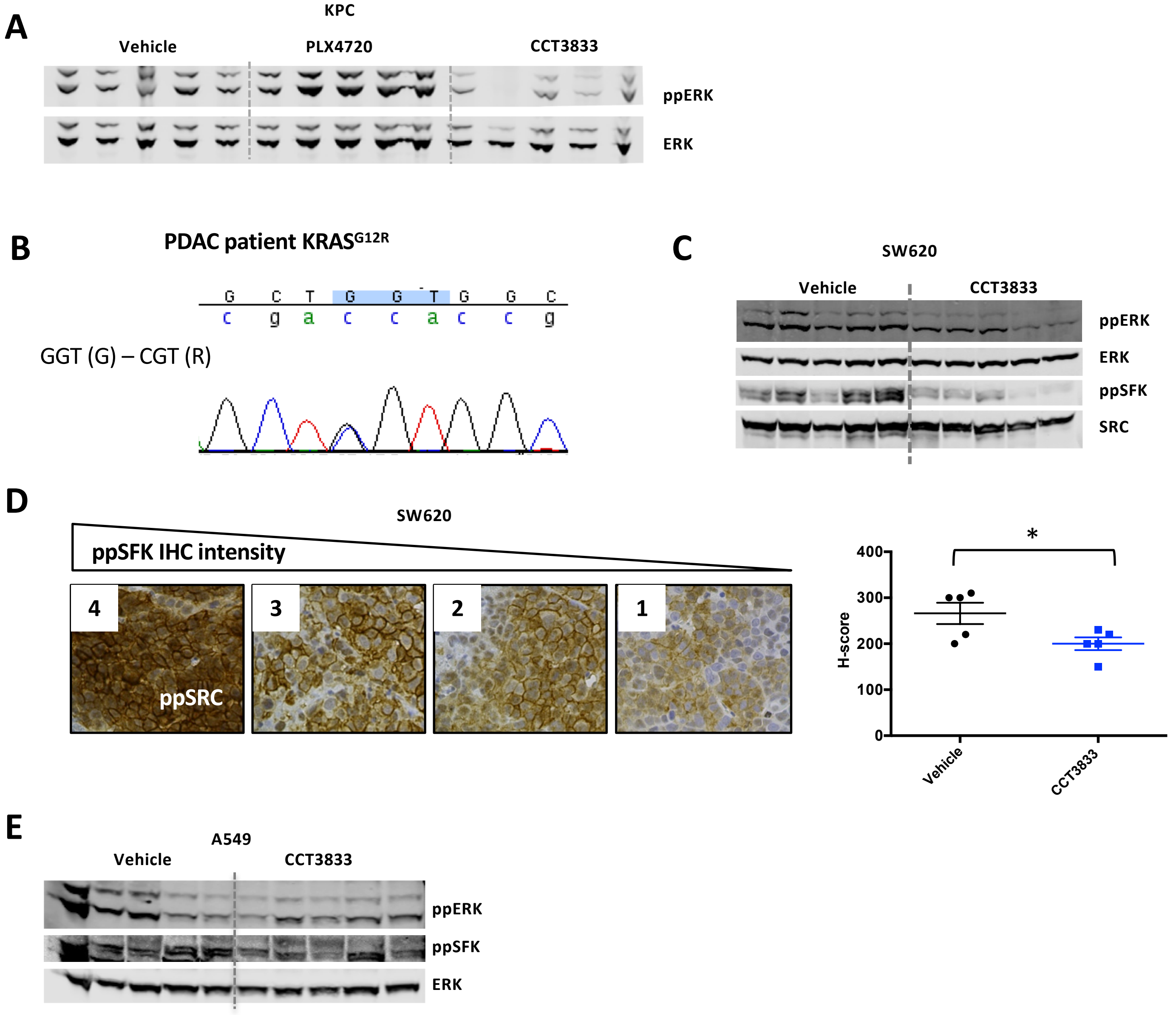

F

A549

Size (mm)	Scoring
<0.5	1
0.5 to 1	2
1 to 2	3
>2	4
>3	5
>4	6
**Foci (No.)**	**Category**
1 to 3	1
3 to 8	2
>8	3

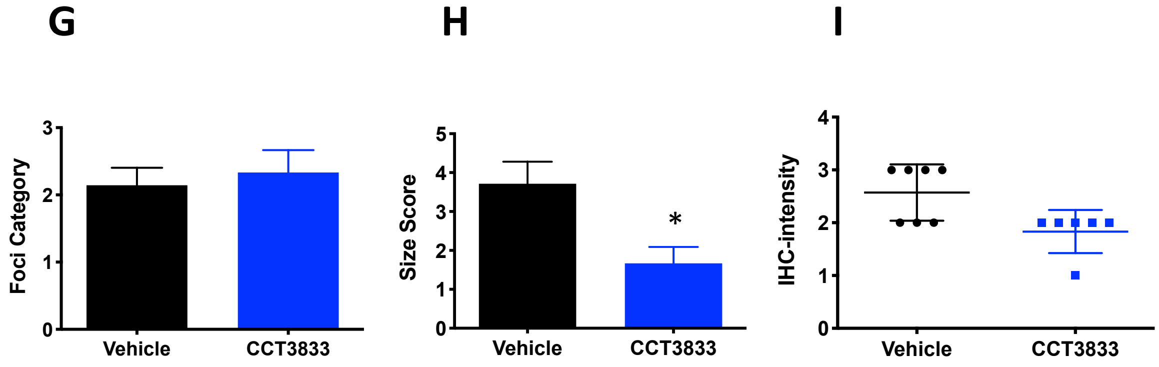

Saturno et al

Supplementary Figure S8

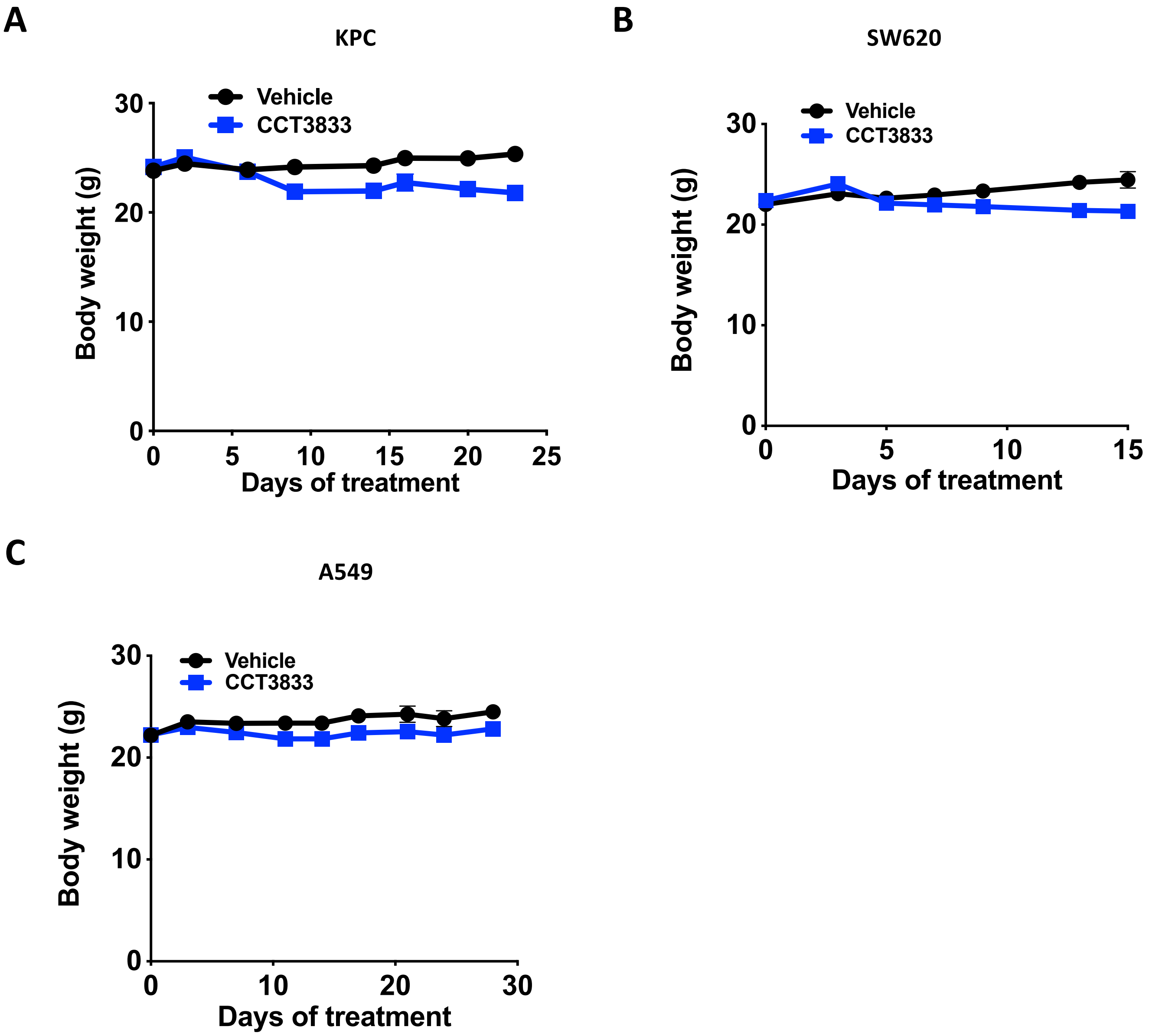

Supplement: Supplementary Figures [file mmc1.pdf]
