# Supplementary material for: The paradox-breaking panRAF plus SRC family kinase inhibitor, CCT3833, is effective in mutant KRAS-driven cancers
Source: Ann Oncol. 2021 Feb;32(2):269–78. doi: 10.1016/j.annonc.2020.10.483 (PMC7839839; doi:10.1016/j.annonc.2020.10.483)
Supplement: Supplementary Information [file mmc2.docx]

**Supplementary Methods**

**Experimental chemistry.** All starting materials, reagents and solvents for reactions were reagent grade and used as purchased. Chromatography solvents were HPLC grade and were used without further purification. Reactions were monitored by thin layer chromatography (TLC) analysis using Merck silica gel 60 F-254 thin layer plates. Flash column chromatography was carried out on Merck silica gel 60 (0.015-0.040mm) or in disposable Isolute Flash Si and Si II silica gel columns. LC-MS analyses were performed on a Micromass LCT / Water’s Alliance 2795 HPLC system with a Discovery 5 μm, C18, 50mm x 4.6mm i.d. column from Supelco at a temperature of 22°C using the following solvent systems: Solvent A: methanol; Solvent B: 0.1% formic acid in water at a flow rate of 1mL/min. Gradient starting with 10% A / 90% B (by volume) from 0-0.5 minutes then 10% A / 90% B to 90% A / 10% B from 0.5 minutes to 6.5 minutes and continuing at 90% A / 10% B up to 10 minutes. From 10-10.5 minutes the gradient reverted back to 10% A / 90% B where the concentrations remained until 12 minutes. UV detection was at 254 nm and ionisation was positive or negative ion electrospray. Molecular weight scan range is 50-1000 m/z. Samples were supplied as 1mg/mL in DMSO or methanol with 3μL injected on a partial loop fill. NMR spectra were recorded in DMSO-d_6_ on a Bruker Advance 500 MHz spectrometer.

3-*tert*-butyl-1-(3-fluorophenyl)-1H-pyrazole-5-carboxylic acid


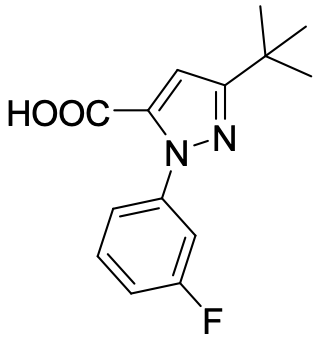


3-fluorophenyl boronic acid (224 mg, 1.6 mmol), ethyl-3-*tert*-butyl-pyrazol-5-carboxylate (320 mg, 1.6 mmol), copper(II) acetate (355 mg, 1.9 mmol) and dry pyridine (158 μL, 1.9 mmol) were suspended under vigorous stirring in an argon atmosphere in 10 mL dry DMF. To the reaction mixture, 4A molecular sieve (300 mg) was added and the suspension stirred for 20 hours at room temperature, then diluted with AcOEt (20 mL), washed with water (2 x 20 mL), then with conc. NaHCO_3_ solution (20 mL), and brine (20 mL), dried (MgSO_4_) and evaporated under vacuum. A thick oil was obtained (520 mg) which was dissolved in EtOH (10 mL), NaOH solution (3 mL, 2M) was added under stirring and the reaction mixture was refluxed for 30 minutes. After cooling to room temperature, the reaction mixture was adjusted to pH 4 (with AcOH) and extracted with AcOEt (20 mL). The organic layer was washed with water (2 x 20 mL), dried and evaporated under vacuum. A solid was obtained (457 mg). The solid was purified on an Isolute Si II column using cyclohexane:AcOEt 3:1 to afford the title compound as a white solid.

Yield 166 mg (39.6% over 2 steps). ^1^H NMR (DMSO-d_6_), δ_H_ (ppm), J (Hz): 1.29 (s, 9H, (CH_3_)_3_C), 6.95 (s, 1H, H_Pyr_), 7.23-7.30 (m, 2H, H_Arom_,_4+5_), 7.35 (d, 1H, H_Arom_,_2_, J=9.8 Hz), 7.44-7.53 (m, 1H, H_Arom,6_), 13.24 (s, 1H, CO_2_H). HRMS: (C_14_H_15_FN_2_O_2_) calcd. 262.1118, found 262.1117.

1{3-*tert*-butyl-1-[(3-fluoro-phenyl)-1*H*-pyrazol-5-yl]}-3-[2-fluoro-4-(3-oxo-3,4-dihydro pyrido[2,3-*b*]pyrazin-8-yloxy)phenyl]urea (CCT3833)


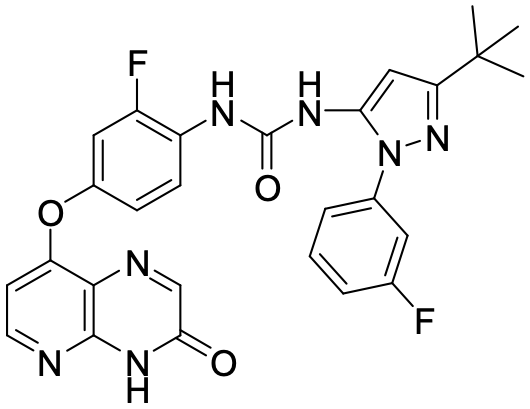


3-*tert*-butyl-1-(3-fluorophenyl)-pyrazole-5-carboxylic acid (81 mg, 0.31 mmol) was dissolved in DMF (2 mL) in a Carousel tube under stirring and inert atmosphere. Then triethyl amine (0.044 mL, 0.32 mmol) and DPPA (0.067 mL, 0.032 mmol) were added and the stirring continued for 30 minutes at 0°C and for an additional 1 hour at room temperature. To this reaction mixture, the 8-(4-amino-3-fluorophenoxy)pyrido[2,3-*b*]pyrazin-3(4*H*)-one (40 mg, 0.15 mmol) was added at once and the tube with the reaction mixture heated at 100°C for 30 minutes, under stirring and an argon atmosphere. After cooling at room temperature, the solution was diluted with 10 mL AcOEt. The organic layer was washed with brine (2 x 10 mL), dried (MgSO_4_), and evaporated to dryness. The residue thus obtained was triturated with Et_2_O and filtered to give the title compound as a pale brown amorphous solid.

Yield: 66 mg (83.0%). ^1^H NMR (500 MHz, DMSO-d_6_) δ: 1.29 (s, 9H, t-Bu), 6.41 (s, 1H, H_Pyrazol_), 6.64 (d, 1H, H_Pyr_, J=5.6Hz), 7.02-7.07 (m, 1H, H_Arom central_), 7.22-7.30 (m, 2H, H_Arom pyrazol_), 7.40-7.44 (m, 2H, H_Arom central_+ H_Arom pyrazol_), 7.53-7.60 (m, 1H, H_Arom pyrazol_), 8.14 (t, 1H, H_Arom central_, J=9.1Hz), 8.17 (s, 1H, H_pyrazinone_), 8.36 (d, 1H, H_pyr_, J=5.6 Hz), 8.87 (s, 1H, NH_urea_), 8.98 (s, 1H, NH_urea_), 12.90 (s, 1H, NH). LC-MS, *t*_R_ = 2.61 min, m/z: 531.2 (M)^+^, calcd for C_27_H_23_F_2_N_7_O_3_. HRMS: (C_27_H_23_F_2_N_7_O_3_), calcd. 531.1830, found: 531.1832.

**Docking studies.** Docking studies were performed using the Maestro suite from Schrodinger (Maestro, version 9.8, Schrödinger, LLC, New York, NY, 2013). We obtained the crystal structures of BRAF (PDB ID: 4JVG) and SRC (PDB ID: 4AGW) in their inactive conformation from the Protein Data Bank (rcsb.org), whereas in case of CRAF a homology model was generated using the BRAF 4JVN structure as template using the Align and Homology modelling routines of MOE version 2016.09. The proteins were prepared using the Protein Preparation Wizard in Maestro and the receptor grid was generated using default settings around the ligand of the original structures. Docking was performed at XP (extra precision) setting and a maximum of 10 docking solutions were stored and re-scored using the strain energy calculation and re-scoring routine of Maestro.

**SRC construct transfection.** HEK-293 and HCT-116 cells were transfected for 48 hours with empty vector, ^wt^SRC or ^T341I^SRC (GenScript) using lipofectamine. To establish stable transfected clones cells were kept under geneticine (1mg/ml) selection.

**Kinase Assays.** Kinase assays were as previously described [1].

**Short-term growth inhibition assays.** Cells were seeded into 96-well plates. 24h later, serial dilutions of CCT3833, PLX4720, TAK-632, ARQ736, MLN-2480, dabrafenib, trametinib or sorafenib were added. Cells were incubated for a further 72-96h and viability measured by CellTiter-Glo assays (Promega) according to manufacturer’s instructions. Relative survival was normalized to control (DMSO = CTL) after background subtraction.

**Long-term cell proliferation assays.** Cells were seeded into 6-well plates with a density between 1500 and 10000 cells/well depending on growth rates. Cells were then treated the following day with the indicated drugs for 10 to 14 days depending on growth rates. Medium was changed every 3 to 4 days. Cellular growth was then determined after removing medium followed by PBS washing and Crystal violet staining. Pictures were then taken for each plate.

**Caspase-Glo luminescence assay.** Cells were seeded into 96-well plates and cultured with the indicated drugs for 24h. The Caspase-Glo 3/7 assay (Promega) was performed according to the manufacturer’s instructions. AU = Arbitrary Units.

***In vitro* tumor spheroids.** 4,000 SW620 cells/well (40 μl/well) were seeded into 96-well hanging drop plates (Sigma) following the manufacturer’s instructions and cultured with the indicated drugs for 5 days. Images were taken at the end of the experiment and analyzed using ImageJ, volume = [(width^2^ × length)/2].

**Histology and immunohistochemistry (IHC).** Tumors were formalin-fixed and prepared for staining with hematoxylin, eosin, and rabbit phospho-SRC (Invitrogen 44660G) or rabbit phospho-ERK (Cell Signaling 20G11) using standard protocols. Positive and negative controls were included in each experiment. The staining scoring of the pattern and intensity was performed in a blinded manner.

**Tissue, cell lysates and immunoblots.**

Tissue and cell lysates were prepared with NP-40 buffer containing, 150mM NaCl, 50mM Tris, pH 7.5, 2mM EDTA, pH 8, 25mM NaF, 1% NP-40, protease inhibitors (Complete, Roche), and Phosphatase Inhibitor Cocktails 2 and 3 (Sigma-Aldrich). All lysates were freshly prepared and resolved by SDS gel electrophoresis for western blotting. Primary antibodies: ERK2 from Santa Cruz (clone C-14); or ppERK1/2 T202/Y204 (clone D13.14.4E, Sigma), ppSFK Y416 (clone D49G4) from Cell Signaling. Specific bands were detected using fluorescent-labeled secondary antibodies (Invitrogen; Li-COR Biosciences) and analyzed using an Odyssey Infrared Scanner (Li-COR Biosciences).

**Mouse allograft/xenografts and *in vivo* efficacy studies.** Female nude mice were injected subcutaneously with 2x10^6^ KPC, 5x10^6^ SW620 cells or 10^7^ A549 cells, tumors were grown to ~100mm^3^, size matched, then mice were randomly allocated to treatment groups using non-statistical methods. Alternatively, 10^6^ A549 luciferase expressing cells (A549-luc-C8, Caliper) were injected into the tail vein of nude mice [2], and at day 67 mice were randomly allocated into treatment groups. PDAC samples for the patient derived xenograft model were collected under the Manchester Cancer Research Centre (MCRC) Biobank ethics application #07/H1003/161+5, with full informed consent from the patients. The work presented in this article was approved by MCRC Biobank Access Committee application 13_RIMA_02. PDAC tumor samples were obtained immediately after surgery. Five to six week old female NOD SCID mice were used for these studies, 6-8 mice per group as indicated. In accordance with our license, animals were excluded if they displayed signs of distress, excessive bodyweight loss (>20%) or illness. This is indicated in Figure 5I with decreasing n numbers, where up to 3 mice were excluded at later time points in the control group. These *in vivo* studies were performed once.

**Mouse xenograft/allograft for *in vivo* pharmacodynamics (PD) studies.** Mice were injected subcutaneously in the right flank with KPC, SW620 or A549 cells grown to 150-200 mm^3^, size matched, then mice randomly allocated to groups of 5. Mice were treated with vehicle control (5% DMSO/water), PLX4720 90 mg/kg or CCT3833 40 mg/kg for 4 days by oral gavage. Tumors were harvested 4h post last dose. These *in vivo* studies were performed once.

**Pharmacokinetics studies.** Drug CCT3833 was suspended in DMSO:water (

**Sanger sequencing.** Tumor gDNA was prepared using Qiagen DNA extraction kit. gDNA was subsequently amplified by PCR and the products were sequenced using dye-terminator chemistry as described [3]. Sequences were visualized using Sequencher software. Oligonucleotide primer sequences are available on request.

**Supplementary Methods References**

1. Niculescu-Duvaz D, Gaulon C, Dijkstra HP et al. Pyridoimidazolones as novel potent inhibitors of v-Raf murine sarcoma viral oncogene homologue B1 (BRAF). J Med Chem 2009; 52: 2255-2264.

2. Jenkins DE, Oei Y, Hornig YS et al. Bioluminescent imaging (BLI) to improve and refine traditional murine models of tumor growth and metastasis. Clin Exp Metastasis 2003; 20: 733-744.

3. Turajlic S, Furney SJ, Lambros MB et al. Whole genome sequencing of matched primary and metastatic acral melanomas. Genome Res 2012; 22: 196-207.

**Supplementary Figure Legends**

**Supplementary Figure S1. CCT3833 *in vitro* selectivity screen.**

**A,** CCT3833 (1µM) efficacy against 114 kinase panel. Color bar: percent activity versus DMSO. **B,** Immunoblot ppERK/ERK, ppSFK/SRC in MIA-PaCa2 cells 4h with DMSO (first lane) or CCT3833 (0.6, 1.2, 2.5, 5, 10µM).

**Supplementary Figure S2. SRC gate-keeper expression in mammalian cells.**

**A,** Representation of the overlap of CCT3833 docking pose in the chicken wild type SRC (pdb code 4AGW) with a model of ^T338I^SRC built from 4AGW using the Maestro suite to introduce a single residue mutation, suggesting a potential steric clash between the large 338I residue and CCT3833 (left). Accordingly, the docking of CCT3833 on the ^T338I^SRC mutant model appears to exclude the drug outside the active pocket (right). **B,** Immunoblot for ppSFK, SRC and GAPDH in HEK-293 cells not transfected (NT), or transfected with lipofectamine (lipo) or empty vector (E.V.) controls, or with human ^wt^SRC or gate-keeper ^T341I^SRC (48h). **C,** Immunoblot for ppSFK, SRC and GAPDH in HEK-293 cells not transfected (NT) or transfected with ^wt^SRC or gate-keeper ^T341I^SRC transfected (48h) and treated with CCT3833 (3833; at 2 or 5µM) for 4 hours. **D,** Immunoblot for ppSFK, SRC and GAPDH in HCT-116 cells not transfected (NT) or transfected with lipofectamine (lipo) control, or with with ^wt^SRC or gate-keeper ^T341I^SRC (48h). **E,** Immunoblot for ppSFK, SRC and GAPDH of HCT-116 cells not transfected (NT) control, or transfected with ^wt^SRC or gate-keeper ^T341I^SRC (48h) and treated with 5µM CCT3833 (3833) for 4 hours.

**Supplementary Figure S3. Proliferation curves, apoptosis and mechanism of action.**

**A-E,** Proliferation of HCT-116 (**A**)**,** SW620 (**B**)**,** MIA-PaCa2 (**C, D**) and Calu-1 (**E**) cells treated with increasing concentrations of the drugs indicated. **F,G** Immunoblot for ppERK, ERK, ppSFK and SRC in Mia-PaCa2 (**F**) and Calu-1 (**G**) cells treated 4h with DMSO (CTL), PLX4720, CCT3833, sorafenib, TAK-632 (all at 1μM), trametinib (20nM). **H,** Caspase 3/7 activation in MIA-PaCa2 cells post DMSO (CTL), CCT3833, TAK-632, PLX4720, sorafenib (all at 1μM), trametinib (20nM), AU = Arbitrary Units, *p≤0.05 student’s t-test. **I,** Long-term proliferation assay on MIA-PaCa2 cells after 10 days of treatment with DMSO (CTL), PLX4720, CCT3833, sorafenib, TAK-632 (all at 1μM), trametinib (20nM). **J,** Bright field images of HCT-116 cells after 48 hours treatment with DMSO (CTL), PLX4720, CCT3833, sorafenib (all at 1μM) or trametinib 20nM.

**Supplementary Figure S4.** **CCT3833 activity in human PDAC cells *in vitro.***

**A, B,** Immunoblot for ppERK, ERK, ppSFK and SRC in HS766T PDAC cells treated with DMSO CTL, PLX4720 1μM, CCT3833 1μM or sorafenib 1μM for 4 hours (**A**) and 48 hours (**B**). **C,** Caspase 3/7 activation in SU86.86 cells after treatment with DMSO (CTL), CCT3833, TAK-632, PLX4720, sorafenib (all at 1μM), or trametinib (20nM) for 24 hours, AU = Arbitrary Units**,** *p≤0.05 student’s t-test**. D,** Long-term proliferation assay on SU86.86 cells, 12 days incubation with DMSO (CTL), CCT3833, or TAK-632 (both at 1μM), or trametinib (20nM).

**Supplementary Figure S5. RAF and SRC dual inhibition is required to efficiently inhibit *KRAS*-mutant cell growth. A,** Long-term proliferation of H23 cells after10 days of DMSO (CTL), CCT3833, TAK-632, saracatinib (all at 1μM), TAK-632 plus saracatinib (TAK+Sara, 1μM each). **B,** Short term cell proliferation assay of A549 and MIA-PaCa2 cells treated with CCT3833 (1.2μM), TAK-632 (1.2μM), bosutinib (2µM) or TAK-632 plus bosutinib (TAK+Bos, 1.2μM+2μM) *p≤0.05 student’s t-test.

**Supplementary Figure S6.** **CCT3833 activity in mouse PDAC cells *in vitro.***

**A,** Short-term proliferation of KPC cells treated with increasing doses of the indicated drugs. **B,** Long-term proliferation assay of KPC 2 cells after 12 days incubation with DMSO (CTL), CCT3833 (1μM), or trametinib (20nM). **C,D,** Caspase 3 and 7 activation in KPC cells from two mice (KPC 1 and KPC 2) after treatment with DMSO (CTL), CCT3833 (1μM), TAK-632 (1μM), PLX4720 (1μM), or trametinib (20nM) for 24 hours. AU = Arbitrary Units, *p≤0.05 student’s t-test.

**Supplementary Figure S7.** **CCT3833 *in vivo* biomarkers**

**A,** Immunoblot for ppERK, ERK in tumors from a biomarker study in KPC allografts after 4 days treatment with vehicle (5% DMSO/water), PLX4720 (90mg/kg) or CCT3833 (40mg/kg). **B,** Sequencing electropherograms confirming *KRAS* mutation in the PDXs from the PDAC patient. The mutated codon is highlighted (blue). **C,** Immunoblot for ppERK, ERK, ppSFK or SRC in SW620 xenografts from biomarker study after 4 days treatment with vehicle (5% DMSO/water) or CCT3833 (40mg/kg). Samples run on the same gel, with dotted line indicating discontinuous regions of the same gel. **D,** ppSFK immunohistochemistry (IHC) in SW620 PD biomarker study**.** IHC and quantification of ppSFK in tumors from the 4-day PD biomarker study, 5 mice/group treated daily for 4 days with vehicle (5% DMSO/water) or CCT3833 (40mg/kg). Images are representative of the scoring: 4 very high; 3 high; 2 medium; 1 low. Quantification of the scoring was calculated according to the H-score formula: (% cells intensity 1*1)+(% cells intensity 2*2) etc. * p ≤ 0.05 student’s t-test. **E,** Immunoblot for ppERK, ppSFK and ERK in A549 xenografts from biomarker study after 4 days treated with vehicle (5% DMSO/water) or CCT3833 (40mg/kg). **F,** Foci number and tumor burden (lesion size) scoring criteria for data presented in **G** and **H** respectively. **G,** Score of number of foci/section assessed by counts of foci on H&E stained sections of individual mouse lungs of A549 tail vein injected mice. **H,** Score of tumor burden in the lungs assessed by measurements of lesions in H&E stained sections of the individual mouse lungs, *p≤0.05 student’s t-test. **I,** IHC scoring of ppERK intensity in lungs of A549 tail vein injected mice.

**Supplementary Figure S8. CCT3833 tolerability.**

**A-C**, Mean body weights of mice treated with vehicle (5% DMSO/water) or CCT3833 (40mg/kg) daily from KPC PDAC (**A**), SW620 (**B**), or A549 (**C**) tumor xenografts efficacy experiments.

**Supplementary Tables**

| **Cell line** | **KRAS status** |
| --- | --- |
| AsPC-1 | G12D |
| MIA-PaCa2 | G12C |
| Capan2 | G12V |
| CF-PAC | G12V |
| HPAC | G12D |
| KPC (mouse) | G12D |
| HS766T | Q61H |
| SU86.86 | G12D |
| HPAF | G12D |
| Panc10.05 | G12D |
| PL45 | G12D |
| PL5 | G12D |
| Panc-1 | G12D |
| H23 | G12C |
| A549 | G12S |
| H2009 | G12A |
| H358 | G12C |
| H-460 | Q61H |
| Calu-1 | G12C |
| HCT116 | G13D |
| SW620 | G12V |
| SW480 | G12V |
| DLD1 | G13D |
| D24 | Wild type |

**Supplementary Table S1: Human Cancer Cell lines and KRAS status.** Panel of *KRAS*-mutant PDAC, CRC and NSCLC cell lines in which CCT3833 was assessed, and their KRAS mutation status.

| **Kinase** | **CCT3833** |
| --- | --- |
| BRAF | 0.420 |
| ^V600E^BRAF | 0.034 |
| CRAF | 0.033 |
| MEK1 | >10 |
| COT | >10 |
| SRC | 0.027 |
| LCK | 0.019 |

**Supplementary Table S2. CCT3833 is a panRAF and SRC family kinase inhibitor.** The table shows the IC_50_ values (μM) of CCT3833 against BRAF, ^V600E^BRAF, CRAF, MEK1, COT, SRC and LCK.


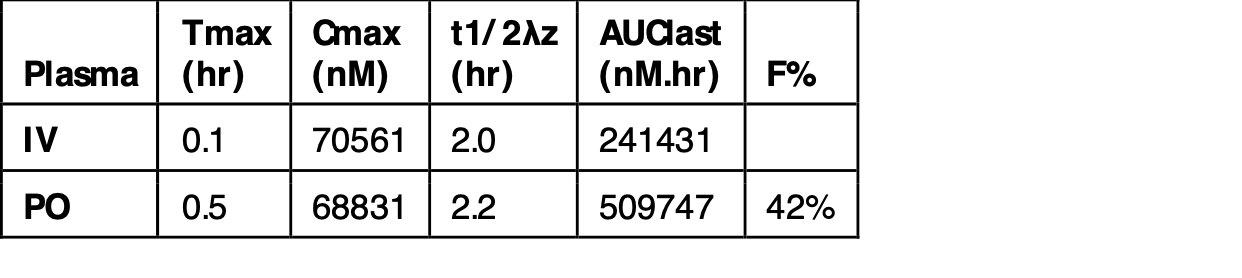


**Supplementary Table S3. Pharmacokinetic parameters for CCT3833 in plasma following oral (10mg/kg) and intravenous (2mg/kg) dosing.**

IV=Intravenous administration, PO=Oral administration, Cmax=maximum concentration, Tmax=time of maximum concentration, AUC(0-t)= area under the concentration time curve, F% =oral bioavailability and t½λz= terminal elimination half life.

| Lesion 1 | Midline subdiaphragmatic nodule |
| --- | --- |
| Lesion 2 | Nodule posterior to gastric antrum |
| Lesion 3 | Mass superior to pancreatic head |
| Lesion 4 | Nodule adjacent to the gallbladder fossa |
| Lesion 5 | Nodule adjacent to the urinary bladder |

**Supplementary Table S4. Patient target lesions measured on scan to determine response to therapy according to RECIST version 1.1 criteria.**
